# Non-prostate cancer tumours: incidence on ^18^F-DCFPyL PSMA PET/CT and uptake characteristics in 1445 patients

**DOI:** 10.1007/s00259-022-05721-z

**Published:** 2022-03-07

**Authors:** Elisa Perry, Arpit Talwar, Sanjana Sharma, Daisy O’Connor, Lih-Ming Wong, Kim Taubman, Tom R. Sutherland

**Affiliations:** 1 Pacific Radiology, Level 1, 123 Victoria Street, Christchurch, Canterbury New Zealand 8013; 2 St. Vincent’s Hospital, Department of Medical Imaging, Melbourne, Victoria Australia; 3 Faculty of Medicine, University of Melbourne, Melbourne, Victoria Australia; 4 St. Vincent’s Hospital, Department of Urology, Melbourne, Victoria Australia; 5 University of Melbourne, Department of Surgery, Melbourne, Victoria Australia

**Keywords:** ^18^F-DCFPyL, PET/CT, PSMA, Prostate cancer, Biochemical recurrence

## Abstract

**Purpose:**

With increasing use of PSMA PET/CT in the staging and restaging of prostate cancer (PCa), the identification of non-prostate cancer tumours (NPCaT) has become an increasing clinical dilemma. Atypical presentations of PSMA expression in prostate cancer and expression in NPCaT are not well established. Understanding the normal and abnormal distribution of PSMA expression is essential in preparing clinically relevant reports and in guiding multidisciplinary discussion and decisions.

**Methods:**

Retrospective review of 1445 consecutive ^18^F-DCFPyL PSMA PET/CT studies by experienced radiologists and nuclear medicine physicians. Lesions indeterminate for PCa were identified. Correlation was made with patient records, biopsy results, and dedicated imaging. Lesions were then categorized into four groups: 1. Confirmed prostate cancer, metastases, 2. NPCaT 3. Benign, and 4. Indeterminate lesions.

**Results:**

68/1445 patients had lesions atypical for prostate cancer metastases. These comprised 8/68 (11.8%) atypical prostate cancer metastases, 17/68 (25.0%) NPCaT, 29/68 (42.6%) indeterminate, and 14/68 (20.6%) benign. In the context of the entire cohort, these are adjusted to 8/1445 (0.6%), 17/1445 (1.2%), 29/1445 (2.0%), and 14/1445 (1.0%) respectively. With the exception of Renal Cell Carcinoma (RCC), NPCaT demonstrated no or low PSMA expression. A similar trend was also observed for indeterminate and benign lesions. Conversely, most atypical PCa metastases demonstrated intermediate or high PSMA expression.

**Conclusion:**

^18^F-DCFPyL PSMA PET/CT detection of NPCaT is low. Lesions demonstrating intermediate to high PSMA expression were exclusively prostate cancer metastases, aside from RCC, and lesions detected in organs with high background expression.

**Supplementary Information:**

The online version contains supplementary material available at 10.1007/s00259-022-05721-z.

## Introduction

Prostate cancer (PCa) is the second most commonly diagnosed cancer in men and is the sixth leading cause of cancer death [1]. Imaging of prostate cancer both at initial staging and at recurrence has been revolutionized by the advent of positron emission tomography (PET) tracers targeted to prostate specific membrane antigen (PSMA) which have shown superiority in comparison with conventional imaging comprising CT and bone scintigraphy [2–4].

PSMA is a transmembrane glycoprotein with high expression in most prostate cancer cells although can be expressed in endothelial cells in non-prostate cancer tumours (NPCaT), particularly in the context of neovascularization [5]. There are several PSMA PET probes available, of which Gallium 68 probes are most widely used. Newer Fluorine 18 probes confer some advantages with longer half-life, opportunity for large scale batch production, and higher target to background resolution. ^18^F-DCFPyL is a commercially available PSMA PET probe used at our institutions.

This wide adoption of PSMA PET/CT with increasing availability of tracers has seen a substantial increase in its use which, along with expanding applications of PSMA in the realms of initial diagnosis, biochemical recurrence, and treatment follow-up, the identification of NPCaT is likely to increase accordingly. The physiological expression of PSMA, expression in benign pathology, and typical patterns of expression in prostate cancer are well documented [6]; however, atypical presentations of PSMA expression in prostate cancer and expression in NPCaT are less established. Understanding the normal and abnormal distribution of PSMA expression is essential in preparing clinically relevant reports and in guiding multidisciplinary discussion and decisions.

Our multicenter international retrospective study is designed to detect the incidence and types of NPCaT detected on ^18^F-DCFPyL PSMA PET/CT in patients with PCa and describe their imaging characteristics. The primary outcome was the incidence of newly diagnosed NPCaT detected in this cohort. We also aimed to evaluate characteristics of atypical prostate cancer metastases and indeterminate lesions. Benign lesions outside the realms of abdominal incidentalomas and incidental lung nodules determined suitable for follow-up protocols were also examined.

## Materials and methods

### Study population

Retrospective multicenter international study using combined data from Pacific Radiology Canterbury, New Zealand (PRC) and St Vincent’s Hospital, Melbourne, Australia (STV). Institutional ethics approval has been granted for the maintenance of a prostate cancer database, from which the study data was derived. Our database includes consecutive patients who have had ^18^F-DCFPyL PET/CT between January 2016 and December 2020. Repeat studies for the same patient were excluded. For patients with multiple studies, only the first showing a suspected NPCaT was included. The patient cohort consisted of patients having a ^18^F-DCFPyL PET/CT for initial staging (35.6%), re-staging (5.1%), and biochemical failure post treatment (59.3%).

### Case selection and imaging analysis

All imaging reports were reviewed to identify patients with suspected incidental NPCaT. Typical prostate cancer-related lesions were defined as PSMA expression greater than background in the expected distribution for prostate cancer within prostate/prostate bed, nodes, bone and visceral locations [6]. Typical sites of nodal involvement include obturator, iliac stations, and retroperitoneum. Although mesorectal nodes have been described as rare or atypical, these were included in the expected distribution as they are increasingly recognised. Distant nodal, liver, and thoracic metastases were also considered typical distributions. Although visceral metastases are described in the absence of nodal or bone involvement, extra-prostatic disease limited to these sites required clarification [6]. These studies were reviewed by either an experienced genitourinary radiologist with subspecialist PET/CT practice or an experienced genitourinary radiologist in consultation with an experienced nuclear medicine physician. Imaging features of the incidental lesions and standardized uptake values (SUVmax) were recorded and categorised according to PROMISE miPSMA expression score. Terminology used in this paper reflecting these guidelines were no expression (below blood pool, score 0), low expression (equal to or above blood pool and lower than liver, score1), intermediate expression (equal to or above liver and lower than parotid gland, score 2) or high expression (equal to or above parotid gland, score 3) [7]. Histology reports were obtained from medical records and pathologic databases, follow-up imaging from the institutional PACS database, and clinical management from the patient’s medical records.

Non-avid incidental lung lesions were assessed by a chest radiologist with > 10 years’ experience. Those less than 10 mm with no PSMA expression and without features suggesting atypical adenomatous hyperplasia/adenocarcinoma spectrum which fitted adopted follow-up guidelines were excluded [8, 9]. Known lesions which had already been identified and investigated on prior imaging were also excluded.

Abdominal ‘incidentalomas’ with no PSMA expression, including adrenal adenomas, liver and renal cysts, were assessed by a subspecialist abdominal radiologist with > 10 years’ experience and those fitting criteria for follow up under ACR white paper for follow-up of incidentalomas were recorded but excluded from end point analysis [10–13].

Patient records were retrieved and subsequent biopsy results, dedicated imaging, multidisciplinary team meeting notes, follow-up clinic letters, and specialist consults were noted. Based on this information in combination with imaging characteristics, lesions were categorized broadly into four groups: 1. confirmed prostate cancer metastases: lesions either in an atypical distribution for PCa and/or considered possible NPCaT, subsequently determined as PCa lesion by histological or clinical confirmation; 2. NPCaT: lesions either in an atypical distribution for PCa and/or considered possible NPCaT, subsequently determined as NPCaT by histological or clinical confirmation; 3. benign: lesions not excluded by lung nodule or incidentaloma criteria either in an atypical distribution for PCa and/or considered possible NPCaT, subsequently determined as benign by histological or clinical confirmation; and 4. indeterminate lesions: lesions not excluded by lung nodule or incidentaloma criteria either in an atypical distribution for PCa and/or considered possible NPCaT, without definitive histological or clinical confirmation. The lesions classified as indeterminate were sub-classified as a. likely benign and b. likely malignant.

### Imaging protocols and reconstruction

^18^F-DCPyL for both centres was sourced from Cyclotek (Melbourne, Australia and Wellington, New Zealand) produced by the same method described previously [14].

PRC: Patients were required to drink 1–2L of water prior to their appointment and void immediately prior to scanning. No diuretics were administered. Patients were imaged on a GE Discovery 690 (General Electric Medical Systems, Milwaukee WI, USA). Low-dose attenuation correction CT images were acquired and reconstructed to 3.75 mm slice thickness with an increment of 3.27 mm using iterative reconstruction (50% ASiR). All patients at both centres were administered 250 MBq (± 50 MBq) of 18F-DCFPyL intravenously in accordance with reference standards outlined by the Australian Radiation Protection and Nuclear Safety Agency (ARPANSA) [15]. Imaging was performed at 120 min (± 10 min) after injection. PET images were acquired at 3.5 min/bed through the pelvis and 3.0 min/bed to the lung apices. Images were reconstructed from time of flight emission data using VUE Point FX and Q-Clear™ “GE Healthcare” iterative technique with a β value of 400. Sharp IR function was applied with no Z-axis filter. PET images were reconstructed on a 256 matrix.

STV: Patients were imaged on a GE Discovery 710 PET/CT (General Electric Medical Systems, Milwaukee WI, USA). Otherwise the scanning protocol matched that described above.

### Statistical analysis

PSMA and pathological findings were assessed using binomial categorical data from unmatched groups compared with a chi-square test. Statistical analyses were conducted with Jamovi software, version 1.2.22.0.

## Results

**Fig. 1:**
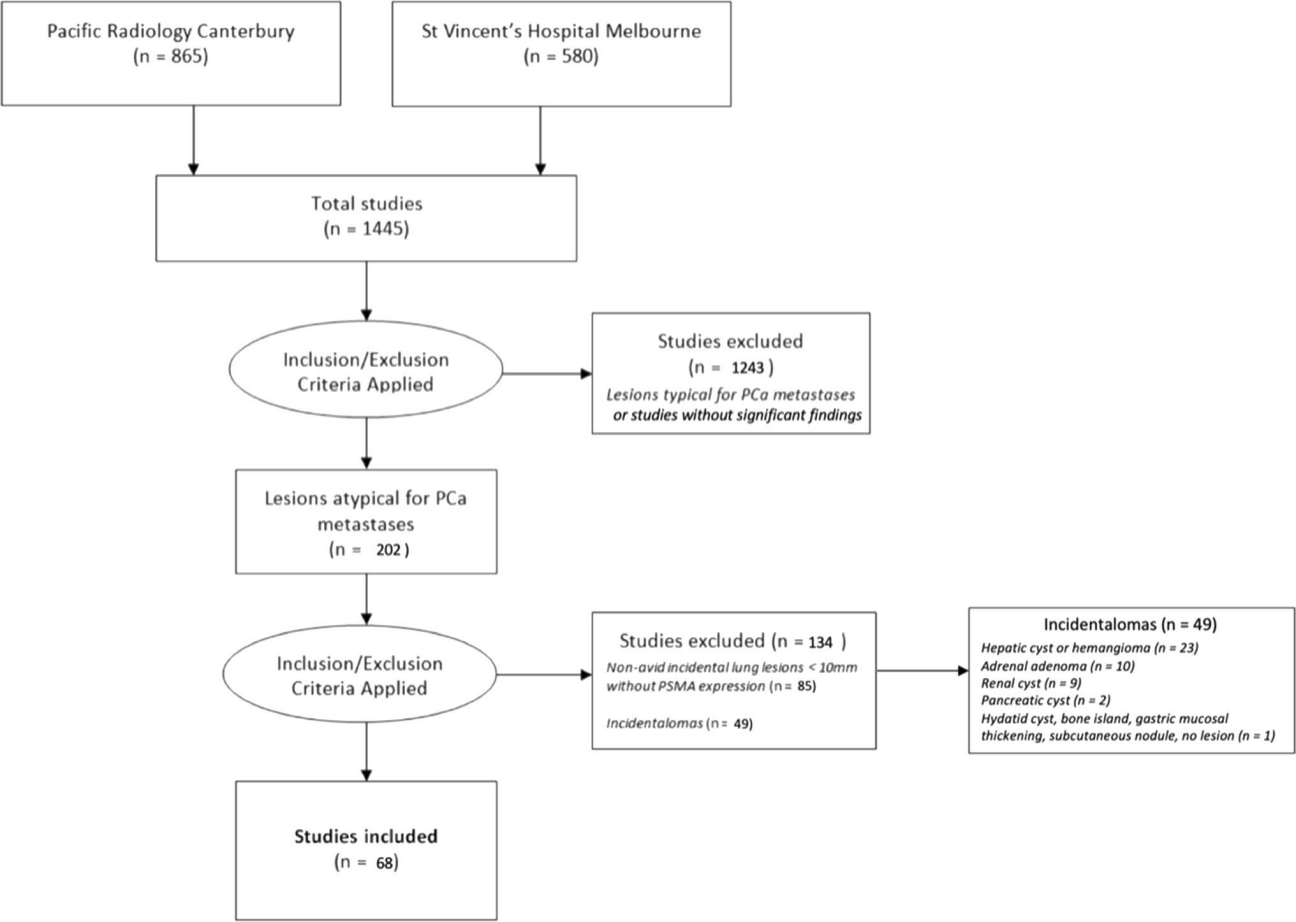
Study selection

A total of 1445 studies were performed using ^18^F-DCFPyL (PRC = 865 studies, STV = 580 studies). One thousand two hundred forty-three of these studies were excluded as they had lesions typical for prostate cancer or no detectable lesion. Two hundred two studies remained for further analysis. Of these studies, 85 related to lung nodules and 49 to incidentalomas, fulfilling the exclusion criteria. Out of 49 incidentalomas, 23/49 (46.9%) were hepatic cysts or hemangiomas, 10/49 (20.4%) were adrenal adenomas, and 9/49 (18.4%) were renal cysts. The remaining 7/49 (14.3%) were made up of pancreatic cysts, subcutaneous nodule, bone island and incidental gastric mucosal thickening. A total of 68 studies were therefore included in our study (Fig. 1).

The remaining 68 lesions comprised 8/68 (11.8%) confirmed prostate cancer metastases, 17/68 (25.0%) NPCaT, 29/68 (42.6%) indeterminate, and 14/68 (20.6%) benign. In the context of the entire cohort, these proportions are adjusted to 8/1445 (0.6%), 17/1445 (1.2%), 29/1445 (2.0%), and 14/1445 (1.0%) respectively.

Within our cohort, the number of false positives included 24/68 (35.3%) patients, who had avid lesions that were proven to be benign either clinically or through biopsy. In the context of the entire cohort, this adjusted to 24/1445 (1.7%) patients.

### Confirmed prostate cancer metastases

5/8 (62.5%) of lesions subsequently confirmed as prostate cancer metastases demonstrated intermediate to high PSMA expression, 4 of which were lung metastases, with biopsy confirmation, and one biopsy confirmed nodal metastasis. The remaining 3/8 (37.5%) lesions were of low or no expression comprising two lung and one bone metastasis demonstrating a range of PSMA expression from SUVmax of < 1 to 5.3 (Table 1).

**Table 1:** Characteristics of confirmed prostate cancer metastases

No	Age	Indication	PSA	Site	Primary SUV	SUV	miPSMA Expression Score	Findings	Clinical Rationale	Outcome
1	74	Biochemical persistence post RP	3.9	Lung	N/A	7.6	2	Solitary LLL nodule 13 mm. No evidence of PCa recurrence elsewhere. Multiple pleural plaques	Morphological appearances suggestive of lung adenocarcinoma lung in increased risk patient without PCa recurrence elsewhere	Biopsy
2	66	BF post RP	0.53	Lung	N/A	11.6	2	Solitary 8 mm RUL lesion, no evidence of PCa recurrence elsewhere	In context of no other sites of recurrence, primary lung cancer should be excluded	Wedge Resection
3	70	BF post RP	0.3	Lung	N/A	22.0	3	High PSMA expression 10 mm LUL nodule. No prostate bed recurrence, equivocal expression in 4 mm left mesorectal node	Equivocal disease elsewhere. Primary lung cancer should be excluded	Resolution of lesion on CT follow up on hormonal therapy
4	71	Initial Staging	11.6	Lung	8.5	11.5	2	21 × 12 mm RUL lobulated solitary nodule in a patient with pulmonary emphysema	No evidence of recurrence elsewhere and significant smoking related lung disease. Primary lung cancer should be excluded	Resection
5	77	Initial Staging	2.6	Lung Bone Node	4.0	< 1.0 6.2 4.0	0	Multiple pulmonary nodules with no PSMA expression, but primary low expression. Low expression enlarged pelvic nodes and sclerotic bone lesions	DDx given as dedifferentiated neuro-endocrine tumour of prostate or metastases from bladder TCC	Lung nodules reduced with Docetaxel and Goserelin
6	60	BF post RP	3.9	Lung	N/A	1.0	0	Multiple new and enlarged pulmonary nodules with low expression, largest 12 × 14 mm RLL apical segment	DDx metastatic PCa versus other malignancy	VATS wedge resection
7	66	BF post XRT	24	Node	N/A	14.1	3	High PSMA expression within left para aortic and left pelvic nodes. ^*^	Recent diagnosis of DLBCL confined to mediastinum. Considered most likely PCa but DLBCL should be excluded	Left para-aortic node excision
8	66	Metastatic PCa on Zoladex, new right pelvic pain	0.4	Bone	45.6	5.3	1	Known multiple PCa bone metastases. New 73 mm expansile lytic right iliac lesion with predominant soft tissue mass, low PSMA expression	Dissimilar appearance to other bony metastases and previous pelvic RT for seminoma, exclude NPCaT	Bone biopsy

*PSA* prostate specific antigen, *SUV* standardized uptake value, *RP* radical prostatectomy, *LLL* left lower lobe, *PCa* prostate cancer, *BCR* biochemical recurrence, *RT* radiotherapy, *RUL* right upper lobe, *PSMA* prostate specific membrane antigen, *LUL* left upper lobe, *CT* computed tomography, *DDx* differential diagnosis, *BPH* benign prostatic hypertrophy, *TCC* transitional cell carcinoma, *RLL* right lower lobe, *VATS* video-assisted thoracoscopic surgery, *DLBCL* diffuse large B cell lymphoma

^*^Although this distribution of nodal involvement is typical for prostate cancer, the recent diagnosis of DLBCL led the MDM to consider a NPCaT, and therefore has been included in this group

### Non-prostate cancer tumours

17/68 (25.0%) patients within our cohort had NPCaT. 2/17 (11.8%) lesions demonstrated intermediate to high heterogeneous PSMA expression and characteristic CT features of renal cell carcinoma (RCC). The remaining 15/17 (88.2%) lesions had no or low PSMA expression. Twelve of these were classified as tumours with high malignant potential and the remaining 3 as low malignant potential.

PSMA and pathological findings of NPCaT in our cohort have been summarized in Table 2. 8/17 (47.1%) of these patients were non-biopsy diagnoses. This was either based on PSMA findings or subsequent imaging displaying characteristic findings of non-prostate cancer; however, in some patients, this diagnosis was made by multidisciplinary consensus as further imaging or biopsy was not felt clinically appropriate due to advanced patient age, performance status or widespread metastatic malignancy.

**Table 2:** PSMA and pathological findings of patients with non-prostate cancer tumours

	Age	Indication	Site	Primary SUVmax	SUVmax	miPSMA Expression Score	Findings	Outcome	Pathology	Malignant Potential	Additional PSMA Findings
1	77	BF	Lung	N/A	3.8	1	29 mm LLL nodule	Biopsy	Primary lung adenocarcinoma	High	Uptake in seminal vesicle and inguinal node
2	79	BF	Lung	29.2	4.8	1	RLL mass	Biopsy	Non-small cell lung cancer	High	Nil
3	73	Post treatment	Kidney	N/A	< 1	0	34 mm right renal lesion	Biopsy	Renal cell carcinoma	High	Nil
4	95	Initial Staging	Kidney	72	19.9	3	78 mm left renal lesion	Clinical	Renal cell carcinoma	High	Nil
5	71	BF	Breast	N/A	2.8	1	10 mm left upper outer lesion	Biopsy	Invasive carcinoma of no special type	High	Nil
6	72	Initial Staging	Pituitary	17.8	1.8	1	Pituitary enlargement	Clinical	Subsequent MRI – pituitary macroadenoma	Low	Uptake within prostate and left superior pubic ramus
7	66	BF	Colon	52.7	4.4	1	Distal transverse colon lesion	Biopsy	Colonic adenocarcinoma	High	Uptake in pre-sacral node
8	81	Initial Staging	Colon	55.6	3.9	1	Ascending colon lesion	Biopsy	Colonic adenocarcinoma and terminal ileum neuroendocrine tumour	High	Uptake in prostate gland
9	63	BF	Colon	N/A	< 1	0	5 cm tubular structure in right iliac fossa	Clinical	Appendix mucocele	Low	Nil
10	64	BF	Brain	N/A	< 1	0	Right posterior temporal lesion	Clinical	Subsequent MRI – Meningioma	Low	Nil
11	64	Initial Staging	Pancreas	6	4.8	1	Dilated pancreatic and bile ducts	Biopsy	Poorly differentiated pancreatic adenocarcinoma	High	Nil
12	59	Initial Staging	Brain	0	4.5	1	Intracranial lesion	Clinical	Subsequent MRI – Glioblastoma	High	Nil
13	77	Initial Staging	Lung	52.7	2.5	1	23 mm RLL nodule	Biopsy	Primary lung adenocarcinoma	High	Uptake in prostate, seminal vesicles, pelvic nodes and bone
14	73	Initial Staging	Kidney	134	4	1	Left upper pole lesion	Clinical	Not investigated due to pre-existing widespread metastatic malignancy	High	Widespread uptake
15	79	Initial Staging	Lymph Node	98.1	3.5	1	24 × 14 mm circumscribed soft tissue lesion posterior to D3	Biopsy	Follicular Lymphoma (cervical node)	High	Uptake in prostate gland
16	70	BF	Lung	0	4.7	1	15 mm left upper lobe nodule	Clinical	Not amenable to biopsy. Likely lung cancer	High	Nil
17	70	BF	Kidney	17.9	10	2	Left renal mass	Clinical	Characteristic for renal cell carcinoma	High	Uptake in pelvic nodes, para-aortic nodes and bone

*SUV* standardized uptake value, *LLL* left lower lobe, *RLL* right lower lobe, *MRI* magnetic resonance imaging, *D3* duodenum (3^rd^ segment), *BF* biochemical failure

9/17 (52.9%) patients had biopsy confirmation. Three of these patients had lung lesions, all of which were biopsy-proven primary lung cancer. Two patients had focal low PSMA expression within the colon, both of which had biopsy-proven colonic adenocarcinoma, one of which had additional biopsies confirming synchronous neuroendocrine tumor within the terminal ileum, occult on PET/CT.

Histopathological assessment of a breast lesion with low PSMA expression (SUVmax 2.8) was proven to be a recurrent ER positive grade 2 breast invasive carcinoma. The remaining three had histopathology consistent with clear cell RCC with no PSMA expression (SUVmax < 1), poorly differentiated pancreatic adenocarcinoma with low PSMA expression (SUVmax 4.8), and follicular lymphoma with low PSMA expression (SUVmax 3.5).

### Indeterminate lesions

25/29 indeterminate lesions demonstrated no or low PSMA expression. 3/29 demonstrated intermediate to high expression but were located in organs with high background expression (liver and spleen) or were secondary to significant inflammation (sinusitis). 1/29 cases demonstrated intermediate expression within the scrotum with repeat imaging demonstrating no interval change over a period of four years. 3/29 (10.3%) were considered most likely prostate cancer metastases without PSMA expression, 7/29 (24.1%) suspicious for NPCaT, and 19/29 (65.5%) were determined most likely benign (Table 3).

**Table 3:** PSMA and pathological findings of patients with indeterminate lesions

	No	Age	Indication	Site	Primary SUV	SUV	miPSMA Expression Score	Findings	Clinical Rationale	Outcome
LIKELY MALIGNANT	1	80	Re-Staging	Node	29.2	1.9	1	Low PSMA expression in left pelvic node. Uptake in left pelvic node.	Known metastatic PCa with bony metastases but no other nodal disease and expression much lower than bone metastases.	Further investigation not pursued due to lesions elsewhere and treated as PCa nodal metastasis
	2	69	Initial Staging	Node	19.2	2.4	1	Uptake in prostate and multiple bilateral prominent iliac nodes up to 12mm, much lower expression than primary.	No confirmation.	Commenced on ADT.
	3	76	BCR post RP	Lung	N/A	1.7	1	11mm ground glass nodule within LUL.	Likely synchronous primary lung Ca.	Follow up CT in 3 months advised. No follow up at STV.
	4	95	Initial Staging	Lung	N/A	2.1	1	Uptake in prostate gland and 19mm spiculated lung nodule in RUL.	Likely synchronous primary lung Ca.	No follow up given age and comorbidities.
	5	72	BF post RP	Lung	49.8	4.2	1	Irregular 14mm pulmonary lesion RUL. Uptake in pelvic nodes.	Likely primary lung adenocarcinoma	No follow up.
	6	83	Re-Staging	Lung	21.4	1.3	1	Uptake in prostate gland and 10mm RLL ground glass pulmonary nodule.	Uncertain significance, possible lung primary.	Stable on follow up CT (4 months). Ongoing follow up.
	7	65	Initial Staging	Skin	N/A	2.1	1	10mm right thigh lesion.	No evidence of primary or metastatic prostate cancer	No follow up as widespread metastases from separate neuroendocrine tumour
	8	75	Re-Staging	Bladder	42.9	N/A*	N/A*	Widespread uptake involving prostate, nodes and right VUJ lesion.	Primary bladder tumour.	No follow up, patient resident abroad and left New Zealand
	9	81	Initial Staging	Lung	26.1	2.7	1	Uptake in prostate, pelvic nodes and low PSMA expression in 11mm nodule within RUL	Likely primary lung adenocarcinoma.	No follow up given comorbidities and age.
	10	73	Initial Staging	Node	26.1	2.1	1	Uptake in prostate, pelvic nodes and low PSMA expression in 14mm mesenteric node	High expression in prostate and pelvic node considered typical for prostate cancer. Mesenteric node indeterminate.	Commenced on ADT with pelvic Radiotherapy. Awaiting further follow-up.
LIKELY BENIGN	1	79	BF post RP	Lung	12.1	2.6	1	Uptake in prostate gland and low PSMA expression in LUL ground glass change	Likely inflammatory.	No follow up.
	2	72	Initial Staging	Lung	17	4.9	1	Uptake in prostate gland and low PSMA expression in LUL ground glass change	Likely inflammatory.	No follow up.
	3	84	BF post RP	Liver	N/A	13.4	3	High PSMA expression within segment 4 of the liver.	Image noise versus liver metastasis, not solid organ disease elsewhere	Not present on follow up PSMA with rising PSA. Most likely benign or artefact.
	4	77	BF post RP	Lung	N/A	2.2	1	Low PSMA expression in 12mm RUL lung nodule[1]	Two sigmoid lesions FDG avid ?metastasis from bowel/prostate or benign lesion	Follow up CT 2 years later showed no significant change in lesion.
	5	69	BF post RP	Lung	N/A	1.6	1	Minimal PSMA expression in 9mm irregular pulmonary nodule	Solitary pelvic node recurrence. Indeterminate lung nodule.	No change on surveillance imaging for over 2 years.
	6	76	BF post RP	Kidney	N/A	<1	0	30mm heterogeneous right retroperitoneal lesion abutting inferior pole of right kidney	Likely benign cyst or lymphatic lesion, exclude sarcoma.	Non-enhancing on dedicated triple phase CT and unchanged over 13 months.
	7	79	BF post RP	Bone	N/A	<1	0	Low PSMA expression in sclerotic left temporal bone lesion.	Likely benign lesion.	No further imaging. Remained asymptomatic.
	8	69	BF post RP	Sinus	N/A	7.5	2	Intermediate PSMA expression in left maxillary sinus mass.	Likely inflammatory, exclude tumour.	Follow up with ENT – CT/MRI demonstrating no suspicious lesion. Changes resolved on imaging 3 years later
	9	70	Initial Staging	Bone	N/A	<1	0	Sclerotic right sacral alar lesion with no PSMA expression, significant expression in primary.	Indeterminate lesion, possibly benign.	FDG PET/CT 2 weeks later demonstrated no avidity. Follow up over 18 months no change
	10	56	BF post RP	Colon	N/A	<1	0	No PSMA expression within sigmoid colon.	Clinical and radiological evidence of diverticulitis.	Resolved. Subsequent PSMA PET/CT no uptake.
	11	83	BF post RP	Lung	N/A	<1	0	No PSMA expression within the lung.	Likely rounded atelectasis.	Resolved on subsequent CT.
	12	74	BF post RP	Larynx	N/A	<1	0	Uptake in seminal vesicle and solid nodule within right false vocal cord.	Likely right laryngocele.	No progression with clinical surveillance.
	13	67	BF post RP	Spleen	N/A	13	3	Pelvic nodal recurrence with low PSMA expression. 7mm hypodense splenic lesion	Indeterminate splenic lesion	Not suitable and patient reluctant for active treatment. Patient remains well over 4 years of clinic follow up.
	14	61	BF post RP	Retro-peritoneal	N/A	<1	0	Thin walled cystic retro-peritoneal lesion.	Most likely benign.	Patient underwent salvage radiotherapy. No specific follow up of retroperitoneal lesion.
	15	63	Initial Staging	Lung	10.8	4.2	1	Uptake in prostate gland and 18mm pleural based nodule	Likely benign.	Resolved on follow up CT 3 months later.
	16	50	Initial Staging	Skin	N/A	3.2	1	Uptake in prostate gland and left paraspinal subcutaneous nodule with low PSMA expression.	Likely benign.	No change on follow up PSMA. No specific comment on follow up regarding skin lesion.
	17	75	BF post RP	Lung	N/A	< 1	0	No PSMA expression in a patchy opacity in LUL.	Likely inflammatory changes.	Follow up CT in 6 weeks advised. No follow up at STV.
	18	73	BF post RP	Thyroid	N/A	1.7	1	Indeterminate heterogeneous 24mm left thyroid nodule	Likely benign nodule.	No follow up.
	19	62	Initial Staging	Scrotum	15.1	5.2	2	Bilateral scrotal extra-testicular nodules	? Epididymal metastases but no extra-prostatic disease elsewhere	Nodules not investigated. Patient proceeded to RP. BF 4 years later with repeat PSMA. No interval changes in scrotal nodules, considered benign

### Benign lesions

Most benign lesions were within the thyroid (6/14) and skin (4/14). 10/14 cases were biopsy proven and 4/14 cases were clinically proven benign lesions. All lesions except a scrotal lesion demonstrated no or low PSMA expression (Table 4).

**Table 4:** PSMA and pathological findings of patients with biopsy or clinically proven benign lesions

No	Age	Indication	Site	Primary SUV	SUV	miPSMA Expression Score	Findings	Clinical Rationale	Outcome
1	65	Initial Staging	Lung	8.9	1.6	1	Uptake in prostate gland and 22 mm lesions within LUL	Suspected bronchogenic malignancy	Biopsy proven granuloma. Reduced in size on follow up imaging
2	72	BF post RP	Lung	25.2	1.3	0	Uptake in pelvic nodes and several pulmonary nodules (most significant 16 mm in RLL)	Suspected benign lesions given low PSMA expression	Wedge resection of RLL lesion confirming Hamartoma
3	77	BF post RP	Skin	6.5	4.5	1	Uptake in abdominal nodes and low PSMA expression in subcutaneous nodules (3 mm and 8 mm)	Direct visualization suggested	Biopsy proven angiolipoma
4	72	BF post RP	Skin	N/A	3.1	1	Low PSMA expression in skin lesion lower right lateral abdomen	Direct visualization suggested	Biopsy performed with non-specific findings, no malignancy
5	65	BF post RP	Skin	N/A	3.0	1	18 mm subcutaneous right paraspinal lesion	Biopsy suggested	Biopsy proven hemangioma
6	68	Initial Staging	Breast	58.3	2.8	1	Low PSMA expression in left breast	Suspected gynaecomastia	Mammogram and biopsy performed confirming gynaecomastia
7	65	BCR post RP	Skin	N/A	1.7	1	Uptake in pelvic nodes and 28 mm rounded lesion deep to skin in right lower back	Probable cyst	Direct visualisation of lesions confirmed sebaceous cyst
8	61	BF post RP	Thyroid	N/A	2.7	1	Multinodular thyroid enlargement causing tracheal narrowing	Probable benign multinodular goitre	Ultrasound confirmation of benign features
9	66	BF post RT	Thyroid	5.8	3.2	1	Indeterminate heterogeneous left thyroid nodule with calcifications	Ultrasound ± FNA suggested	Biopsy proven benign thyroid nodule
10	57	BF post RP	Thyroid	N/A	2.6	1	38 × 28 mm ovoid homogeneous mass in lower pole of left thyroid lobe	Ultrasound ± FNA suggested	Biopsy proven benign thyroid nodule
11	69	BF post RP	Thyroid	5.5	< 1	0	No PSMA expression in a 40 mm nodule within the thyroid isthmus	Ultrasound suggested	Ultrasound confirmation of benign features
12	66	BF post RT	Thyroid	3.5	4.6	1	Indeterminate heterogeneous left thyroid nodule with calcifications	Ultrasound ± FNA suggested	Biopsy proven benign thyroid nodule
13	70	BF post RP	Thyroid	N/A	2.3	1	25 mm heterogeneous density nodule in right thyroid with calcifications	Ultrasound suggested	Ultrasound confirmation of benign features
14	58	BF post RP	Scrotum	N/A	7.8	2	Unilateral right scrotal extra-testicular nodule with PSMA expression	? Epididymal metastases but no recurrence elsewhere	Orchidectomy pre-salvage, histology showed granulomatous epididymitis

*SUV* standardized uptake value, *LUL* left upper lobe, *BF* biochemical failure, *RP* radical prostatectomy, *RLL* right lower lobe, *PSMA* prostate specific membrane antigen

## Discussion

This study represents the largest cohort to date assessing incidence of NPCaT detected by PSMA imaging and is the only study exclusively examining this incidence with ^18^F-DCFPyL PET/CT. PSMA imaging is considered highly specific for prostate cancer although this specificity is only realized in combination with a comprehensive knowledge of the physiological and abnormal expression of PSMA. Physiological expression and distribution of typical prostate cancer related abnormal expression is well documented. [6]

Atypical PCa metastases are seen in less than 5% of cases but can affect most organs. Atypical metastases are rare in isolation and are often observed in the context of a typical pattern of disseminated metastatic PSMA expressing PCa. In addition, PCa metastases are described as focal with high PSMA expression whereas NPCaT expression is more likely to be low and non-focal [6, 16]. All lesions in our cohort categorized as PCa metastases were in expected sites for metastatic disease but NPCaT required exclusion due to their structural features or clinical presentation (Table 1). The majority of lesions confirmed to be PCa metastases demonstrated intermediate to high PSMA expression, with two cases of multiple lung lesions demonstrating no expression. This echoes the study by Damjanovic et al. which concluded that 27.5% of prostate cancer metastases demonstrated no PSMA expression. (Damjanovic 2018) Our study demonstrated that lesions with intermediate to high PSMA expression were more likely to be PCa metastases rather than NPCaT regardless of their CT morphology. All of the NPCaT in our group (except for two RCC cases) demonstrated no or low PSMA expression (SUV < 5). These findings correlate with literature describing PSMA expression in RCC [17, 18]. Although some cases in our cohort were not followed up due to factors including patient age, comorbidity, and extensive tumour burden, many lesions were subject to MDM discussion, clinical and radiological follow-up, and/or biopsy. This approach is valid and necessary in the clinical workup of these patients particularly in the context of advancing treatment options for patients with oligometastatic disease.

Numerous benign lesions are also known to express PSMA; however, from our cohort, the indeterminate and benign lesions largely demonstrated no or low PSMA expression (SUVmax < 5) [16, 19]. Pulmonary nodules in this patient cohort were common and the majority were assigned to follow-up based upon established guidelines [8, 9]. Lung nodules comprised the majority of the incidental potentially malignant group although these were larger (11–40 mm) with more complex imaging features and some demonstrated low PSMA expression. We found that lung nodules with intermediate or high PSMA expression were exclusively PCa metastases in our cohort whereas no biopsy-proven lung cancer demonstrated intermediate or high PSMA expression, despite PSMA expression in lung cancer described in the literature [20]. Our study has demonstrated that PCa metastases are substantially more frequent than NPCaT in the context of thoracic lesions with intermediate to high PSMA expression. These findings are further substantiated when considered in the context of existing structured reporting systems. For example, the European Association of Nuclear Medicine, including authors of both PROMISE data and PSMA-RADS, has recently provided guidelines for standardised reporting using E-PSMA five-point scale. The majority of the indeterminate and NPCaT lesions in our cohort comply with category 3 E-PSMA (indeterminate) lesions and the majority of benign lesions correspond to category 2 E-PSMA (likely benign). Furthermore, many lesions later confirmed to be PCa metastases arguably fell under E-PSMA 5, which would correctly allocate them to PCa metastases, but additional findings beyond this definition prompted clinical uncertainty, such as morphology, solitary site of disease, and other malignancy and predisposing factors for second primary [7, 21, 22].

The ability to differentiate PCa metastases from NPCaT is vital as further investigation can lead to morbidity, delays in therapy and incurs additional medical costs. In our cohort, 8% of patients with benign incidental findings underwent a biopsy as part of further investigation while of 19 patients with lung nodules over 10 mm, 13 (68%) were biopsied. Recognizing these patterns in context of established standardised reporting criteria can give PET/CT specialists the ability to make a confident diagnosis, thus avoiding escalating investigation, cost and therapeutic delays [7]. Importantly we would emphasise that guidelines and structured reporting systems allow for reduced variation of interpretation and clear communication however overall interpretation critically relies upon multiple factors and a multidisciplinary approach to diagnosis and management is paramount [7, 22].

The incidence of NPCaT in our PSMA cohort (1.7%) is substantially less than the incidence of significant incidental non-FDG avid findings on FDG PET/CT (22.6%) [23]. There are a number of potential reasons for this, including differing demographics, definitions of ‘major’ clinical significance, stricter evidence-based criteria used in our study, the use of subspecialist radiologists to exclude benign pathologies along with our exclusion of pre-existing known pathologies.

PSMA expression in NPCaT is more commonly associated with tumours which undergo neovascularization such as RCC, breast, glial tumours, gastrointestinal, pancreatic and lung tumours, all of which were represented in our cohort [24–29]. Further tumours reported to express PSMA not represented in our study include oral SCC, salivary ductal carcinoma, medullary thyroid carcinoma, small cell lung cancer, osteosarcoma, gynaecological malignancies, and adenoid cystic tumours [30, 31]. Such expression is variable but has significant clinical implications. PSMA imaging may provide an investigative tool for such tumours, with particular recent interest in clear cell RCC and in detection and characterisation of metastatic diseases [18, 32–35]. The potential for PSMA targeted radiopharmaceuticals in non-prostate tumours is vast and the degree of PSMA expression may prospectively select treatment candidates and monitor response. Treatment monitoring, in particular drugs targeting neovascularization, e.g. bevacizumab and tyrosine kinase inhibitors, is a further potential application. PSMA expression in NPCaT may aid prognostication, for example, PSMA expression in non-metastatic triple negative breast cancer confers worse prognosis with higher relapse and reduced response to androgen receptor inhibition [25, 30]. In contrast, PSMA expression in non-small cell lung cancer (NSCLC) is associated with earlier stage tumours. It is noteworthy that these concepts remain in the realm of research and the full clinical impact of these applications is yet to be determined [24, 36].

This study benefited from a large number of consecutive patients in a multicenter international setting. A limitation of this study was its retrospective design. The largest impact of this was that many patients did not have histological confirmation and/or did not have conclusive follow up, leading to indeterminate findings in a cohort of patients. Selection of patients based on initial reports can introduce subjectivity and bias; however, the initial reports were generated by subspecialty trained experienced radiologists and nuclear medicine physicians. The imaging centers used different scanners albeit two consecutive generations of the same product, however this may have affected SUVmax measurements. Low numbers of individual non-prostate cancer tumours limit the ability to provide specific recommendations. There is always a degree of subjectivity when categorizing the significance of incidental findings and no perfect system exists although we have attempted to mitigate this by using experienced subspecialist radiologists and by considering the opinion of multidisciplinary meetings.

## Conclusion

Our work is the largest study to date examining incidence of NPCaT detected by PSMA PET/CT and is the only study exclusively examining incidence in ^18^F-DCFPyL PET/CT. PSMA imaging of PCa is highly specific with the detection of PSMA expressing NPCaT exceedingly rare. NPCaT in our cohort generally demonstrated low or no PSMA expression. Although PSMA expression was noted in RCC, this was lower and less focal than typical PCa metastatic disease. We found that significant PSMA expressions at sites typical for prostate cancer metastases were exclusively PCa metastases rather than NPCaT.

## Supplementary Information

Below is the link to the electronic supplementary material.

Supplementary file1 (DOCX 4432 KB)

## Data Availability

At request.

## References

[1] Culp MB, Soerjomataram I, Efstathiou JA, Bray F, Jemal A (2020). Recent global patterns in prostate cancer incidence and mortality rates. Eur Urol.

[2] Morris MJ, Rowe SP, Gorin MA, Saperstein L, Pouliot F, Josephson D, et al. Diagnostic Performance of 18F-DCFPyL-PET/CT in Men with Biochemically Recurrent Prostate Cancer: Results from the CONDOR Phase III, Multicenter Study. Clinical Cancer Research. 2021. 10.1158/1078-0432.CCR-20-4573 PMC8382991 33622706

[3] Pienta KJ, Gorin MA, Rowe SP, Carroll PR, Pouliot F, Probst S (2021). A Phase 2/3 Prospective Multicenter Study of the Diagnostic Accuracy of Prostate Specific Membrane Antigen PET/CT with 18F-DCFPyL in Prostate Cancer Patients (OSPREY). J Urol.

[4] Tanaka T, Yang M, Froemming AT, Bryce AH, Inai R, Kanazawa S, et al. Current Imaging Techniques for and Imaging Spectrum of Prostate Cancer Recurrence and Metastasis: A Pictorial Review. RadioGraphics. 2020:190121. 10.1148/rg.2020190121 32196428

[5] Conway RE, Rojas C, Alt J, Nováková Z, Richardson SM, Rodrick TC (2016). Prostate-specific membrane antigen (PSMA)-mediated laminin proteolysis generates a pro-angiogenic peptide. Angiogenesis.

[6] Barbosa FG, Queiroz MA, Nunes RF, Viana PC, Marin JFG, Cerri GG (2019). Revisiting prostate cancer recurrence with PSMA PET: atlas of typical and atypical patterns of spread. Radiographics.

[7] Eiber M, Herrmann K, Calais J, Hadaschik B, Giesel FL, Hartenbach M (2018). Prostate cancer molecular imaging standardized evaluation (PROMISE): proposed miTNM classification for the interpretation of PSMA-ligand PET/CT. J Nucl Med.

[8] Baldwin DR, Callister ME (2015). The British Thoracic Society guidelines on the investigation and management of pulmonary nodules. Thorax.

[9] MacMahon H, Naidich DP, Goo JM, Lee KS, Leung AN, Mayo JR (2017). Guidelines for management of incidental pulmonary nodules detected on CT images: from the Fleischner Society 2017. Radiology.

[10] Berland LL, Silverman SG, Gore RM, Mayo-Smith WW, Megibow AJ, Yee J (2010). Managing incidental findings on abdominal CT: white paper of the ACR incidental findings committee. J Am Coll Radiol.

[11] Gore RM, Pickhardt PJ, Mortele KJ, Fishman EK, Horowitz JM, Fimmel CJ (2017). Management of incidental liver lesions on CT: a white paper of the ACR Incidental Findings Committee. J Am Coll Radiol.

[12] Herts BR, Silverman SG, Hindman NM, Uzzo RG, Hartman RP, Israel GM (2018). Management of the incidental renal mass on CT: a white paper of the ACR Incidental Findings Committee. J Am Coll Radiol.

[13] Mayo-Smith WW, Song JH, Boland GL, Francis IR, Israel GM, Mazzaglia PJ (2017). Management of incidental adrenal masses: a white paper of the ACR Incidental Findings Committee. J Am Coll Radiol.

[14] Perry E, Talwar A, Taubman K, Ng M, Wong L-M, Booth R, et al. [18 F] DCFPyL PET/CT in detection and localization of recurrent prostate cancer following prostatectomy including low PSA< 0.5 ng/mL. European Journal of Nuclear Medicine and Molecular Imaging. 2021;48:2038–46. 10.1007/s00259-020-05143-9 33399941

[15] ARPANSA. Nuclear Medicine Diagnostic Reference Levels (DRLs). Nuclear Medicine Diagnostic Reference Levels (DRLs). Australian Government. 1999. https://www.arpansa.gov.au/sites/default/files/nuclear-medicine-diagnostic-reference-levels.pdf. Accessed: 7 December 2021.

[16] Hofman MS, Hicks RJ, Maurer T, Eiber M (2018). Prostate-specific membrane antigen PET: clinical utility in prostate cancer, normal patterns, pearls, and pitfalls. Radiographics.

[17] Pozzessere C, Bassanelli M, Ceribelli A, Rasul S, Li S, Prior JO (2019). Renal cell carcinoma: the oncologist asks, can PSMA PET/CT answer?. Curr Urol Rep.

[18] Rowe SP, Gorin MA, Hammers HJ, Javadi MS, Hawasli H, Szabo Z (2015). Imaging of metastatic clear cell renal cell carcinoma with PSMA-targeted 18 F-DCFPyL PET/CT. Ann Nucl Med.

[19] Kirchner J, Schaarschmidt BM, Sawicki LM, Heusch P, Hautzel H, Ermert J (2017). Evaluation of practical interpretation hurdles in 68Ga-PSMA PET/CT in 55 patients: physiological tracer distribution and incidental tracer uptake. Clin Nucl Med.

[20] Schmidt LH, Heitkötter B, Schulze AB, Schliemann C, Steinestel K, Trautmann M (2017). Prostate specific membrane antigen (PSMA) expression in non-small cell lung cancer. PLoS One..

[21] Ceci F, Oprea-Lager DE, Emmett L, Adam JA, Bomanji J, Czernin J (2021). E-PSMA: the EANM standardized reporting guidelines v1. 0 for PSMA-PET. Eur J Nucl Med Mol Imaging..

[22] Rowe SP, Pienta KJ, Pomper MG, Gorin MA (2018). PSMA-RADS version 1.0: a step towards standardizing the interpretation and reporting of PSMA-targeted PET imaging studies. Eur Urol..

[23] Sheldon JA, Yap KK, Taubman KL, Schlicht SM (2018). Prevalence of non 18F-fluorodeoxyglucose-avid incidental findings of clinical significance on whole body positron emission tomography/computed tomography: A review of 500 consecutive cases. J Med Imaging Radiat Oncol.

[24] Farag M, Bolton D, Lawrentschuk N (2020). Prostate-specific membrane antigen for the surgical oncologist: interpreting expression beyond the prostate. ANZ J Surg.

[25] Kasimir-Bauer S, Keup C, Hoffmann O, Hauch S, Kimmig R, Bittner A-K (2020). Circulating tumor cells expressing the prostate specific membrane antigen (PSMA) indicate worse outcome in primary, non-metastatic triple-negative breast cancer. Front Oncol.

[26] Malik D, Kumar R, Mittal BR, Singh H, Bhattacharya A, Singh SK (2018). 68Ga-labeled PSMA uptake in nonprostatic malignancies: has the time come to remove “PS” from PSMA?. Clin Nucl Med.

[27] Patel DN, Karsh LI, Daskivich TJ. Next-generation imaging in localized high-risk prostate cancer. Nature Publishing Group; 2021. 10.1038/s41391-021-00356-x 33790419

[28] Shetty D, Patel D, Le K, Bui C, Mansberg R (2018). Pitfalls in gallium-68 PSMA PET/CT interpretation—a pictorial review. Tomography.

[29] Stoykow C, Huber-Schumacher S, Almanasreh N, Jilg C, Ruf J (2017). Strong PSMA Radioligand Uptake by Rectal Carcinoma: Who Put the" S" in PSMA?. Clin Nucl Med.

[30] Fragomeni RAS, Amir T, Sheikhbahaei S, Harvey SC, Javadi MS, Solnes LB (2018). Imaging of nonprostate cancers using PSMA-targeted radiotracers: rationale, current state of the field, and a call to arms. J Nucl Med.

[31] Sharma P (2020). 68Ga-PSMA-avid small cell lung cancer on PET/CT – Incidental second malignancy in treated prostate cancer. Clin Nucl Med.

[32] Chang SS, Reuter VE, Heston W, Gaudin PB (2001). Metastatic renal cell carcinoma neovasculature expresses prostate-specific membrane antigen. Urology.

[33] Fragomeni RAS, Menke JR, Holdhoff M, Ferrigno C, Laterra JJ, Solnes LB (2017). Prostate-specific membrane antigen–targeted imaging with [18F] DCFPyL in high-grade gliomas. Clin Nucl Med..

[34] Rhee H, Blazak J, Tham CM, Ng KL, Shepherd B, Lawson M (2016). Pilot study: use of gallium-68 PSMA PET for detection of metastatic lesions in patients with renal tumour. EJNMMI Res.

[35] Yin Y, Campbell SP, Markowski MC, Pierorazio PM, Pomper MG, Allaf ME (2019). Inconsistent detection of sites of metastatic non-clear cell renal cell carcinoma with PSMA-targeted [18 F] DCFPyL PET/CT. Mol Imag Biol.

[36] Backhaus P, Noto B, Avramovic N, Grubert LS, Huss S, Boegemann M (2018). Targeting PSMA by radioligands in non-prostate disease—current status and future perspectives. Eur J Nucl Med Mol Imaging.

