# Supplementary material for: Non-prostate cancer tumours: incidence on 18F-DCFPyL PSMA PET/CT and uptake characteristics in 1445 patients
Source: Eur J Nucl Med Mol Imaging. 2022 Mar 7;49(9):3277–88. doi: 10.1007/s00259-022-05721-z (PMC9250467; doi:10.1007/s00259-022-05721-z)
Supplement: Supplementary file 1 — Supplementary file1 (DOCX 4432 KB) [file 259_2022_5721_MOESM1_ESM.docx]

***NON-PROSTATE CANCER TUMOURS: INCIDENCE ON ^18^F-DCFPyL PSMA PET/CT AND PSMA EXPRESSION CHARACTERISTICS IN 1445 PATIENTS.***

**European Journal of Nuclear Medicine and Molecular Imaging**

***Electronic Supplementary Material***

Authors

Elisa Perry (1,2,3), Arpit Talwar (2), Sanjana Sharma (2), Daisy O’Connor (1), Lih-Ming Wong (4,5), Kim Taubman (2), Tom R Sutherland (2,3)

1.   Pacific Radiology, Christchurch, Canterbury, New Zealand

2.   St. Vincent’s Hospital, Department of Medical Imaging, Melbourne, Victoria, Australia

3.   Faculty of Medicine, University of Melbourne, Melbourne, Victoria, Australia

4.   St. Vincent’s Hospital, Department of Urology, Melbourne, Victoria, Australia

5.   University of Melbourne, Department of Surgery, Melbourne, Victoria, Australia

Corresponding Author

Elisa Perry MBChB, BSc, MRCP, FRCR, FRANZCR

Radiologist

Pacific Radiology, Level 1, 123 Victoria Street, Christchurch, Canterbury, New Zealand 8013

Telephone: +64 3 374 9546

Email: [elisa.perry@pacificradiology.com](mailto:elisa.perry@pacificradiology.com)

**Fig. S1**

**Prostate Cancer Solitary Metastasis to Lung**

71 year old presenting for initial staging of prostate cancer. Axial CT reconstructed on lung windows (a) acquired as the CT component of the ^18^F-DCFPyL PSMA study demonstrates a 21mm solitary pulmonary nodule in the right upper lobe (arrow) and background smoking related lung disease. Fused axial PET/CT reconstruction (b) demonstrates significant PSMA expression, SUVmax 11.5, within the lesion (arrow head). As there was no evidence of nodal or metastatic disease typical for PCa elsewhere and the patient had a significant smoking history, the differential was of PSMA expression in a primary lung cancer versus a solitary pulmonary PCa metastasis. Histology following wedge resection confirmed metastatic prostate adenocarcinoma.


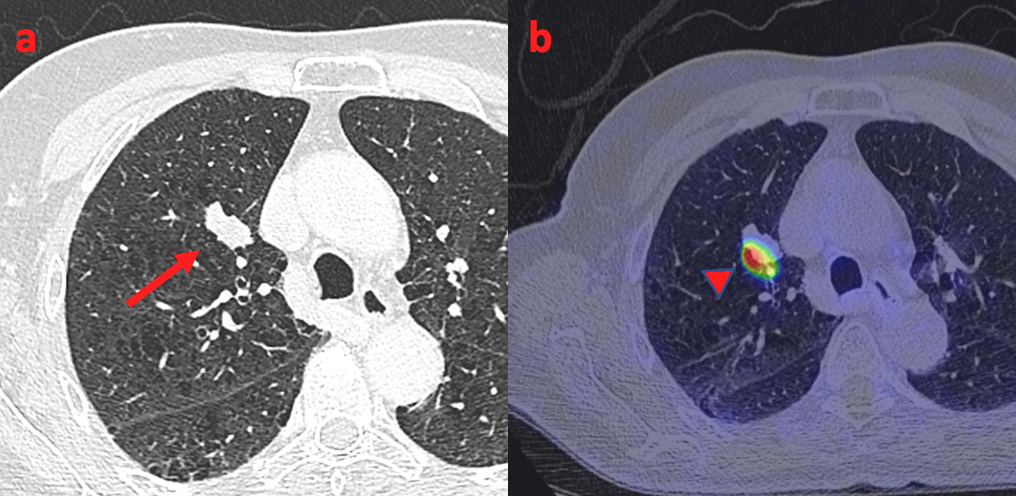


**Fig. S2**

**Primary Lung Cancer**

77 year old presenting with biochemical recurrence of prostate cancer. Axial CT reconstructed on lung windows (a) acquired as the CT component of the ^18^F-DCFPyL PSMA study demonstrates a 23mm mixed density pulmonary nodule in the right lower lobe (arrow). Fused axial PET/CT reconstruction (b) demonstrates minimal PSMA expression within the lesion, SUVmax 2.5 (arrow head). Widespread significant PSMA expression in abdominal and pelvic lymph nodes with additional lesion consistent with bone metastasis. Differential was of low PSMA expression in pulmonary PCa metastasis versus primary lung cancer. Histology following CT guided biopsy confirmed primary lung adenocarcinoma.


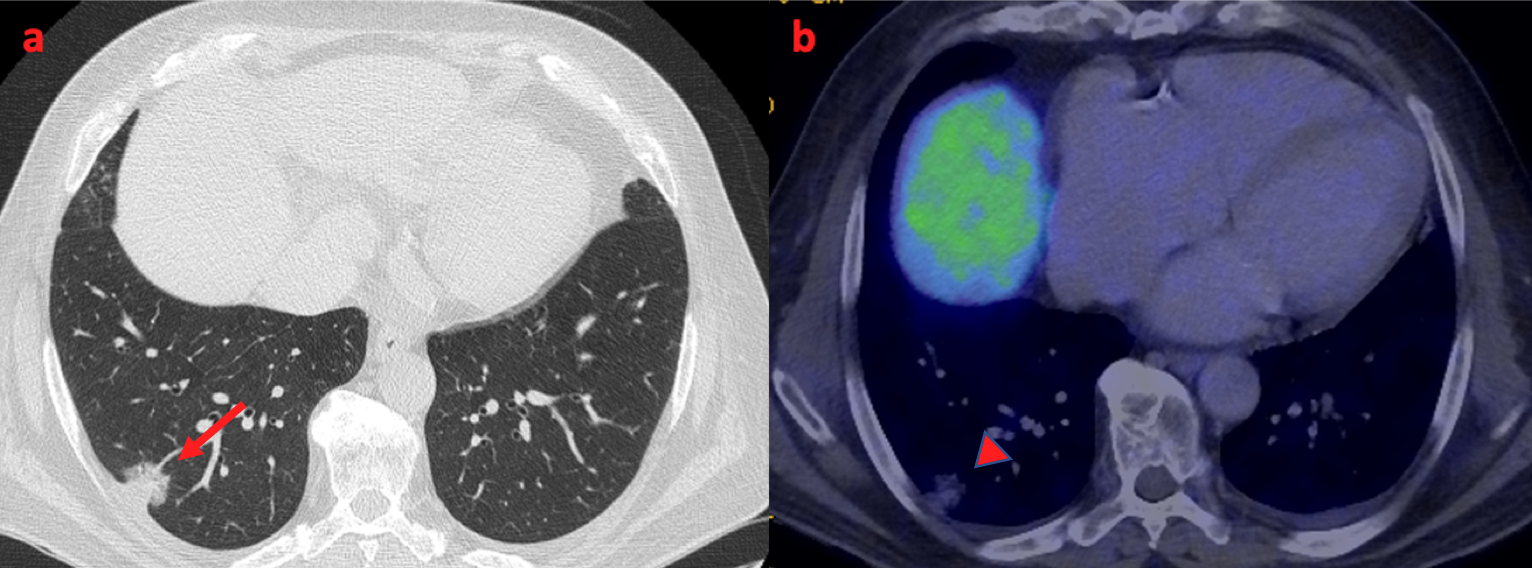


**Fig. S3**

**Renal cell carcinoma**

95 year old presenting for initial staging of prostate cancer. Coronal CT reformat acquired as the CT component of the ^18^F-DCFPyL PSMA study demonstrates a 78mm complex mass arising from the left kidney (arrow). Fused coronal (b) and axial (d) PET/CT reconstruction and axial inverted TOF images (c) demonstrate heterogeneous PSMA expression with some foci demonstrating significant expression, SUVmax 19.9, (arrow head). Due to patient age and comorbidity, no histological confirmation was obtained.

***
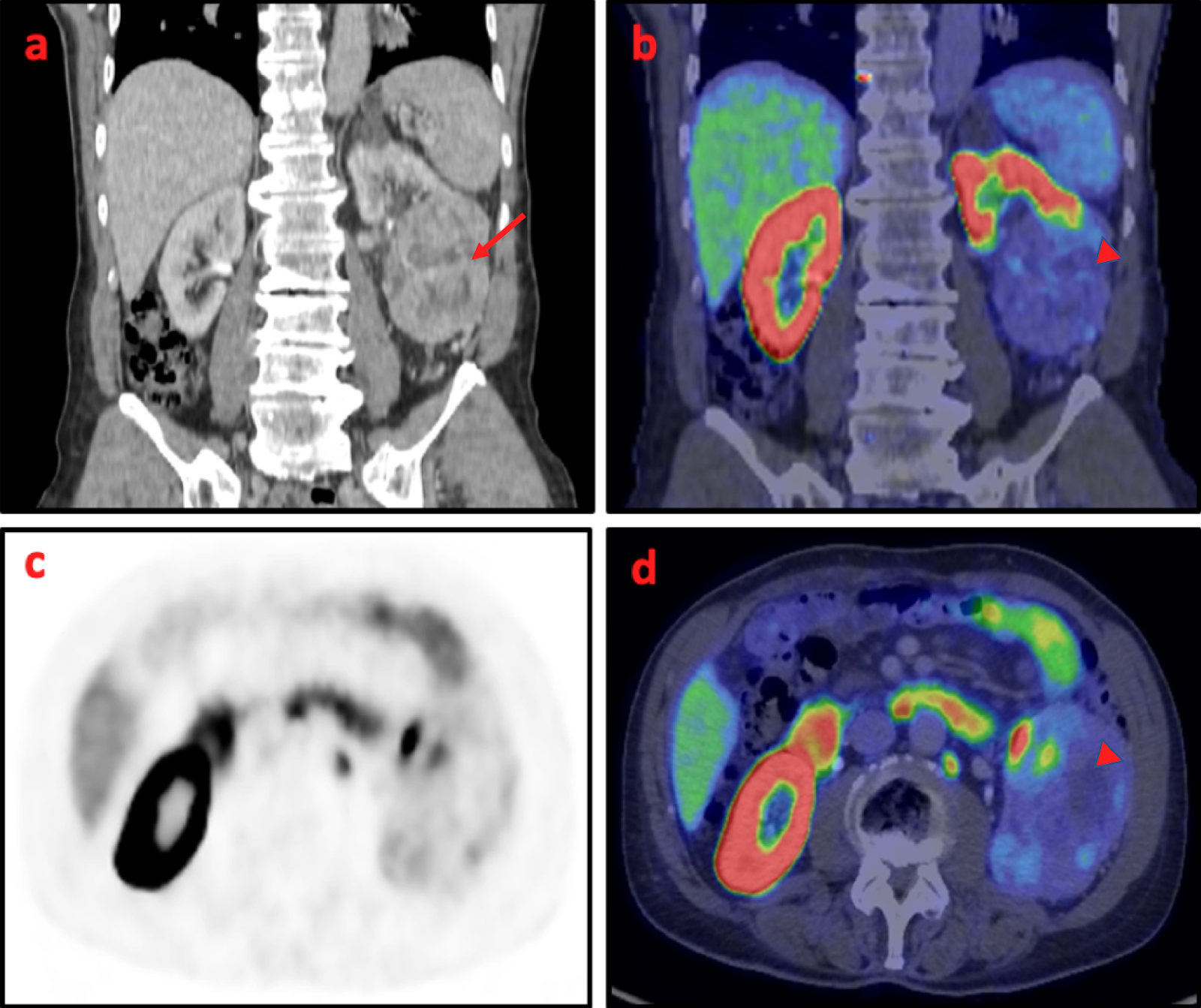
***

**Fig. S4**

**Colonic adenocarcinoma with gastrocolic fistula**

66 year old presenting for initial staging of prostate cancer. Axial reconstruction from contrast medium enhanced CT (a) acquired as the CT component of the ^18^F-DCFPyL PSMA study demonstrates mural thickening centered on the distal transverse colon (arrows). Coronal reformats (b) demonstrate associated gastrocolic fistula (arrow heads). Axial Inverted Time of Flight (TOF) images (c) and fused PET/CT (d) demonstrate low PSMA expression, SUVmax 4.4 (hollow arrows). Histology confirmed colonic adenocarcinoma.


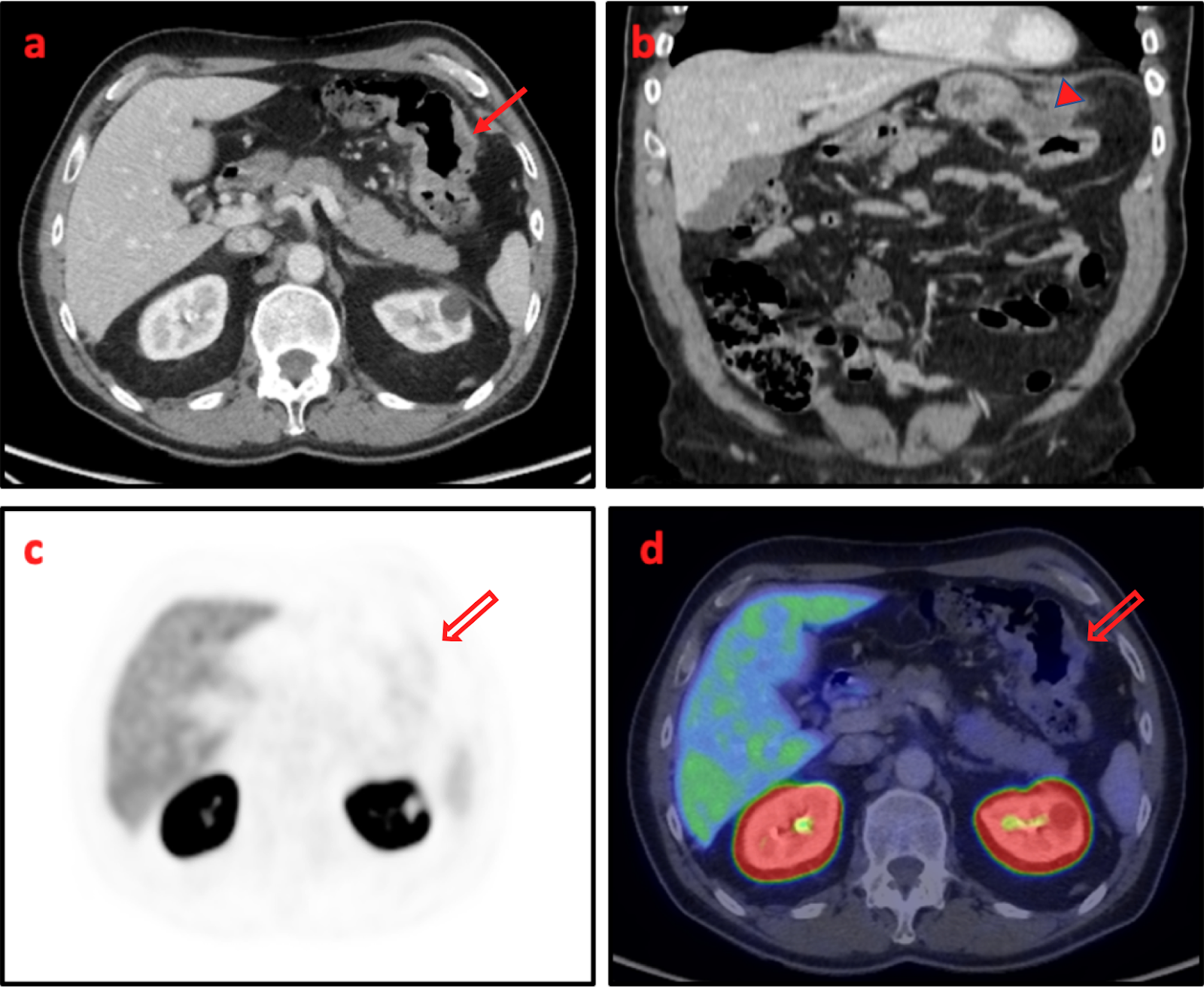


**Fig. S5**

**Breast carcinoma**

71 year old presenting for initial staging of prostate cancer. Axial CT reformat acquired as the CT component of the ^18^F-DCFPyL PSMA study demonstrates a 10mm lesion on the left upper breast/inferior axillary region (arrow). Fused axial PET/CT reconstruction (b) demonstrates minimal PSMA expression, SUVmax 2.8 (arrow head). Biopsy confirmed primary breast cancer.

***
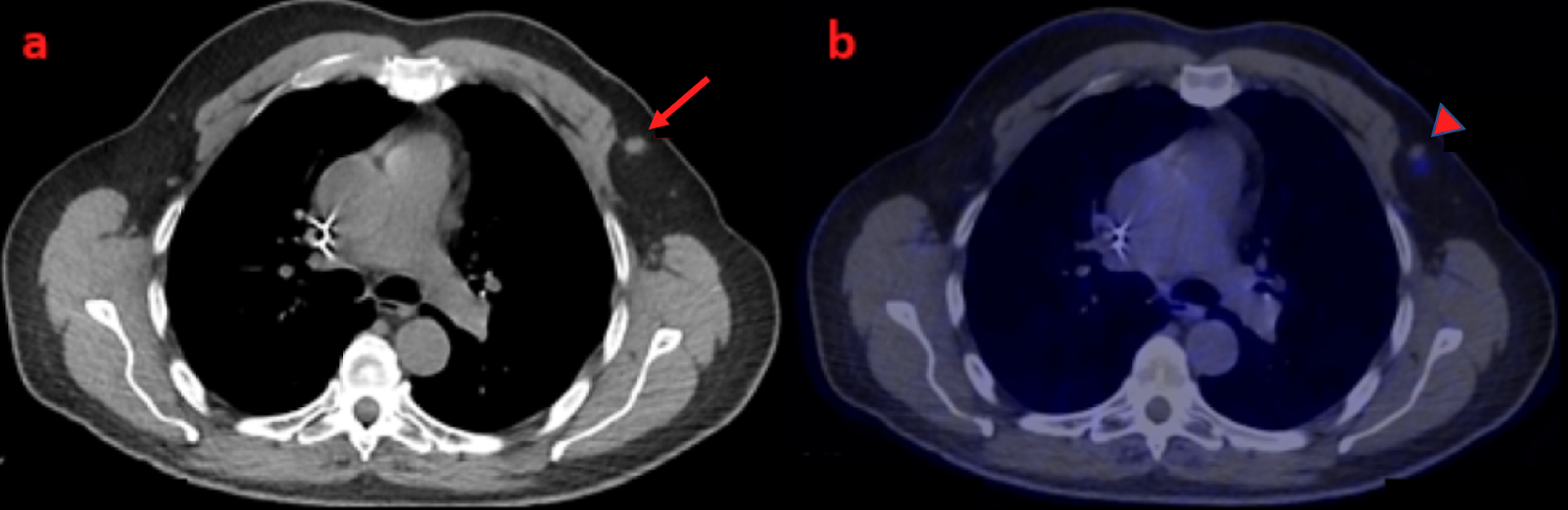
***
